# Radionecrosis and cellular changes in small volume stereotactic brain radiosurgery in a porcine model

**DOI:** 10.1038/s41598-020-72876-w

**Published:** 2020-10-01

**Authors:** Hamed Zaer, Andreas Nørgaard Glud, Bret M. Schneider, Slávka Lukacova, Kim Vang Hansen, John R. Adler, Morten Høyer, Morten Bjørn Jensen, Rune Hansen, Lone Hoffmann, Esben Schjødt Worm, Jens Chr. Hedemann Sørensen, Dariusz Orlowski

**Affiliations:** 1 Centre for Experimental Neuroscience (CENSE), Department of Neurosurgery, Aarhus University Hospital, Palle Juul-Jensens Boulevard 165, indgang J, Plan 1, J118-125, (Krydspunkt 116), 8200 Aarhus N, Denmark; 2 Department of Clinical Medicine, Aarhus University, Aarhus, Denmark; 3 Zap Surgical Systems, Inc., San Carlos, CA USA; 4 Danish Center for Particle Therapy, Aarhus University Hospital, Aarhus, Denmark; 5 Department of Neurosurgery, Stanford University School of Medicine, Stanford, CA USA; 6 Department of Psychiatry and Behavioral Sciences, Stanford University School of Medicine, Stanford, CA USA; 7 Department of Oncology and Radiation Therapy, Aarhus University Hospital, Aarhus, Denmark; 8 Department of Nuclear Medicine and PET Center, Institute of Clinical Medicine, Aarhus University and Hospital, Aarhus, Denmark

**Keywords:** Experimental models of disease, Translational research, Neuroscience

## Abstract

Stereotactic radiosurgery (SRS) has proven an effective tool for the treatment of brain tumors, arteriovenous malformation, and functional conditions. However, radiation-induced therapeutic effect in viable cells in functional SRS is also suggested. Evaluation of the proposed modulatory effect of irradiation on neuronal activity without causing cellular death requires the knowledge of radiation dose tolerance at very small tissue volume. Therefore, we aimed to establish a porcine model to study the effects of ultra-high radiosurgical doses in small volumes of the brain. Five minipigs received focal stereotactic radiosurgery with single large doses of 40–100 Gy to 5–7.5 mm fields in the left primary motor cortex and the right subcortical white matter, and one animal remained as unirradiated control. The animals were followed-up with serial MRI, PET scans, and histology 6 months post-radiation. We observed a dose-dependent relation of the histological and MRI changes at 6 months post-radiation. The necrotic lesions were seen in the grey matter at 100 Gy and in white matter at 60 Gy. Furthermore, small volume radiosurgery at different dose levels induced vascular, as well as neuronal cell changes and glial cell remodeling.

## Introduction

Performing neurosurgery with minimum injury to the surrounding healthy brain tissue, and beyond that modulation of neurocircuits, especially in the deep brain structures without interfering with other normal brain functions, has always been an issue in every neurosurgical approach. Restoration of neuronal functions or damaged neural circuitries has however only been considered in recent decades^1^. The invention of radiosurgery in the 1950s by Lars Leksell was primarily intended to substitute the neurosurgical knife with converging narrow beams of ionizing radiations, the so-called Gamma knife (GK), to minimize complications of open surgery^2^. Shortly thereafter the GK was used in functional brain surgery for targeting deep fiber tracts or nuclei, and this opened the door to a new world in the field of functional neurosurgery for non-invasive treatment of Parkinson disease, psychiatric disorders, and intractable pain. The intended clinical effects of these procedures were linked to the “interruption of the neuronal pathways” due to the creation of ablative lesions^3^. A notable exception was the treatment of trigeminal neuralgia, during which Leksell observed that the mechanism of action involved changes that barely could be explained in his view by cell death concerning the decrease in pain intensity and frequency right after radiosurgery^4,5^. This trend led to other studies of the dose–effect association suggesting a different mechanism of cell death in radiosurgery, namely death by nuclear damage and reproductive disruption, in contrast to the interphase cell degeneration by cytoplasmic membrane lesions^6–9^. Moreover, radiosurgery of epileptogenic AVMs in functional areas has shown cessation or remission of epileptic attacks, unexpectedly prior to fully obliteration of AVM^10^. This observation led to the idea of using a radiosurgical approach to treat medically refractory mesial temporal lobe epilepsy (MTLE) without the existence of space-occupying lesion, instead of surgical resection of the temporal lobe^11^. An early effect of radiosurgery was also seen by the decrement of seizure attacks right after irradiation without concomitant neuroimaging changes during the first 6 months^10^. The first transient changes in MRI with gadolinium were observed after 6 months, which were no longer present at 9 months; although, suddenly after 10 months the changes reappeared on both MRI and positron emission tomography (PET)^11,12^. These observations led to animal studies on the dose–response of normal and pathological brain tissue to radiation^13,14^. Similarly, behavioral and histological changes were seen simultaneously in rats targeted in striatum^15^. Furthermore, transcriptomic studies on GK radiated striatum done in rats, have shown a different level of changes in irradiated and even non-irradiated ipsilateral and contralateral sides, which confirm the dose-dependent biological effect of radiosurgery^16^.

It has been shown that the delivery of sub-lethal doses of radiation at a specifically targeted node within brain circuits, alters their function^14^. However, the basic cellular mechanism of GK Surgery is still unknown despite the advances in the usage of focal cerebral irradiation in the treatment of functional brain disorders^15,17^. Ablative radiosurgery is based on animal studies to define the dose, volume, and tissue response to irradiation. The absence of lesions or minimal changes in the latest neurophysiological, radiological and histological studies in both animals and humans despite the observed clinical effects have been interpreted lately against the formerly suggested “lesional effect’’^17–20^. However, this absence could be due to the lack of sufficient imaging or histology procedures that would detect apoptosis, permeability changes due to acute endothelial cell loss or edema. In general, the survival of tissue during ionizing radiation depends on the specific cell type, the volume of the tissue, and the intensity of the delivered doses^21^. Therefore, the present study aimed to establish a porcine model for investigating the dose-dependent tissue reaction of ultra-high single radiation doses to very small volumes of the cerebral white and grey matter in a large animal. Evaluation of safety and efficacy of different high radiation doses in small volumes of the brain in the large animal models is a necessary step to determine the tissue response inducing focal changes in the brain activity without causing necrotic changes. The Göttingen minipig has been established as a large animal model, which is an ethically acceptable alternative to primate models, and it furthermore allows more specific radiosurgical targeting due to the larger pig brain compared to rodents^22,23^.

## Results

### MRI and FDG-PET/CT

**Figure 1:**
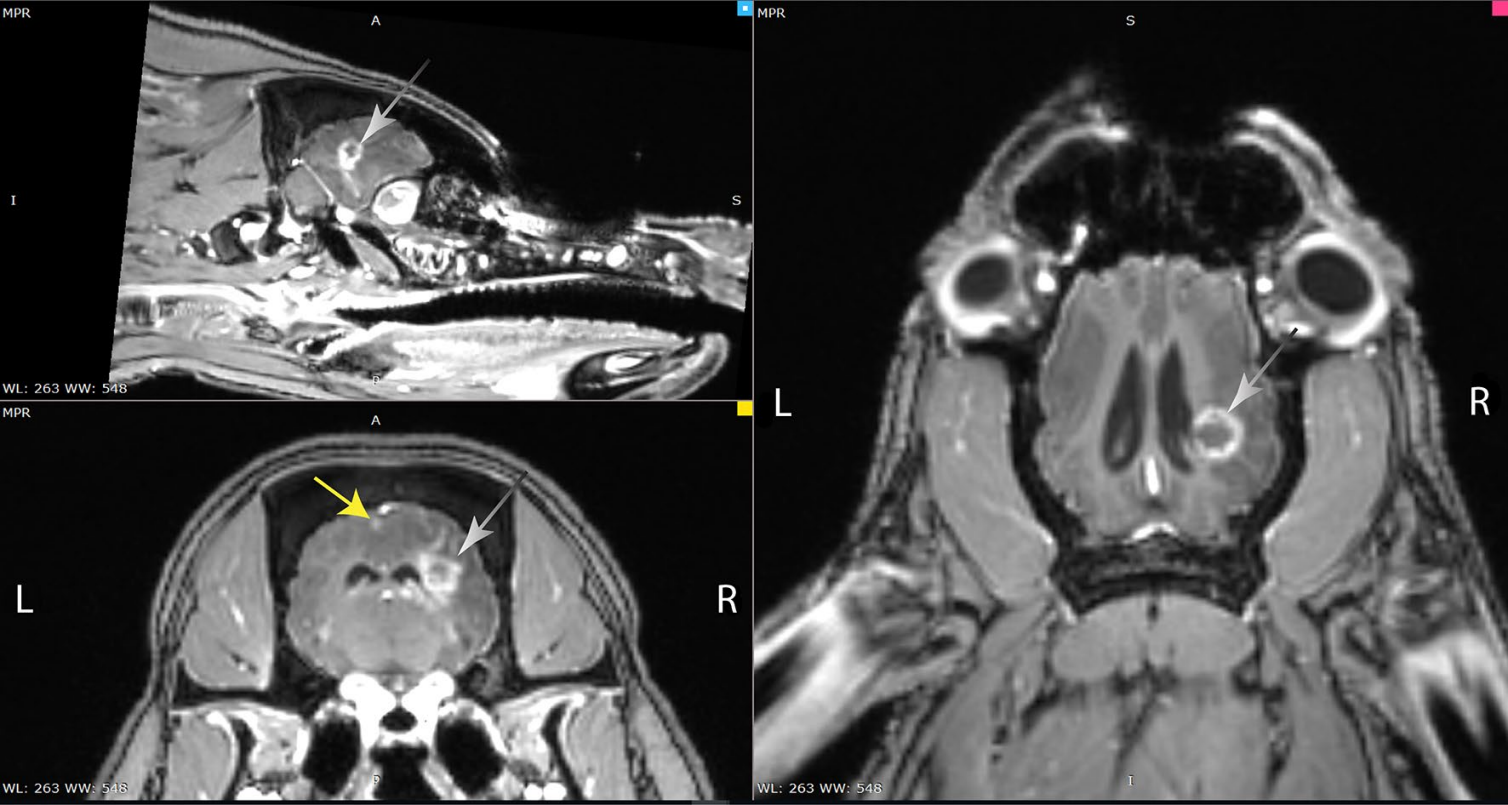
MRI (T1) with Gadoteric acid contrast showing changes in the grey (yellow arrowhead) and white matter (white arrows) of the brain from animal A03/100 Gy, five months after irradiation.

MRI changes were observed as increased uptake of Gadoteric acid in the aimed white matter area of both animals with 100 Gy after five months (Fig. 1). Increased uptake of the contrast was seen in the white matter after irradiation by both 5 and 7.5 mm aperture. However, changes in the motor cortex were just visible in the A03/100 Gy/5 mm aperture. No changes were seen in the other animals in the first six months. The animals, which had received doses of 80 and 60 Gy, showed the primary sign of increased Gadoteric acid uptake in the white matter after seven months, without any changes in the cortex. The diameter of the changes increased with the radiation dose, as can be seen in Table 1.

**Table 1:** Results summary.

Peripheral radiation dose-aperture	Motor cortex		Internal capsule	
	MRI	Histology	MRI (Necrosis volume)	Histology
0 Gy	–	–	–	–
40 Gy–5 mm	–	–	–	–
60 Gy–5 mm	–	–	83.4 ± 8.4 mm^3^	Radio-necrosis
80 Gy–5 mm	–	–	260.8 ± 20.6 mm^3^	Radio-necrosis
100 Gy				
5 mm	Increased gadolinium uptake	Necrosis	259.3 ± 15.4 mm^3^	Radio-necrosis
7.5 mm	–	Condensed Chromatin	330.3 ± 9.1 mm^3^	Radio-necrosis

**Figure 2:**
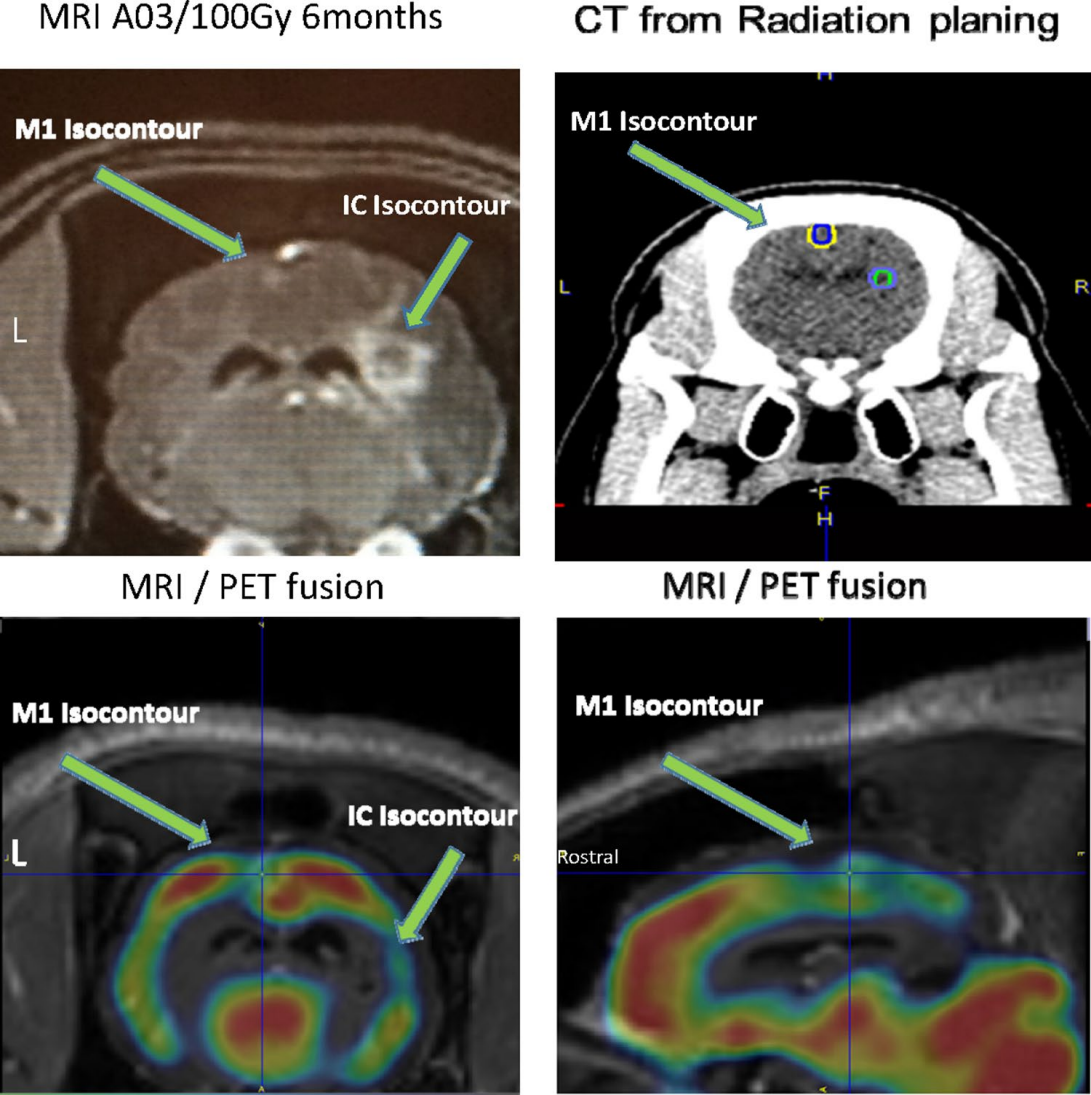
(**A**) MRI changes in animal A03/100 Gy after 6 months. (**B**) Radiation planning CT scan from animal A03/100 Gy. (**C**,**D**) MRI/PET fusion after six months in animal A03/100 Gy.

We did not see any certain systematic changes in PET signal in the irradiated minipigs. However, when the MRI showed tissue changes (Fig. 2A) we also retrogradely found an asymmetry in the PET-FDG signals in the VOI of the IC and M1 lesions after MRI/PET fusion (Fig. 2C,D). These changes were seen 6 months post-irradiation, and only in the animals irradiated with 100 Gy. White matter decrease of FDG/PET uptake was observed in both irradiated targets with 5 and 7.5 mm aperture; however, cortical changes were detectable in the animal irradiated via a 5 mm aperture in the cortex.

### Histology of motor cortex

**Figure 3:**
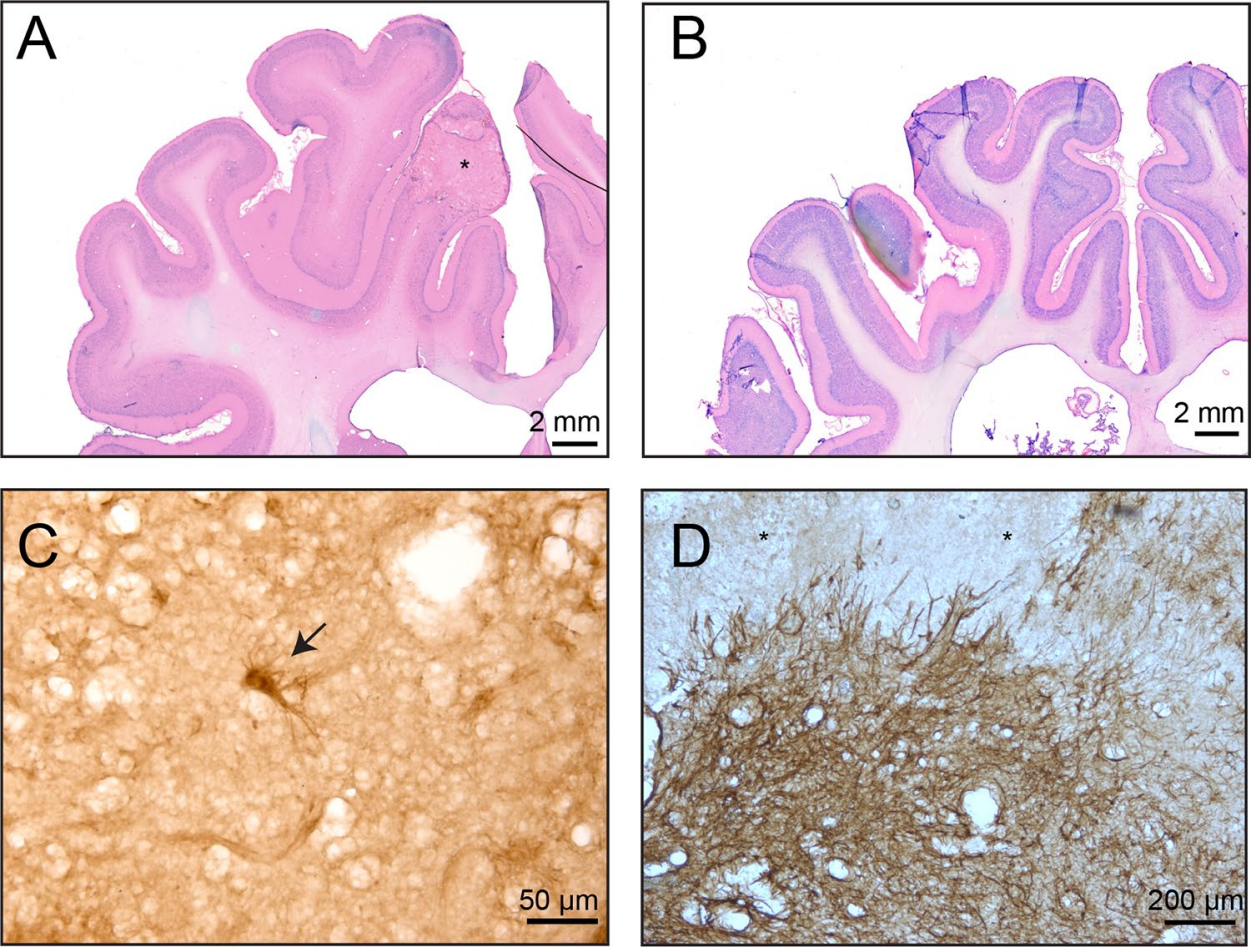
(**A**) Animal 03/100 Gy/5 mm, Motor cortex, Nissl&Eosin, *necrotic area. (**B**) Animal 04/80 Gy, Nissl&Eosin, no visible necrotic change. (**C**) Animal 03/100 Gy/5 mm anti_Caspase-3, arrow—possible Caspase 3 positive neuron, (**D**) Animal 03/100 Gy/5 mm, anti-GFAP, a visible dispersed glial scar in the motor cortex, *necrosis.

**Figure 4:**
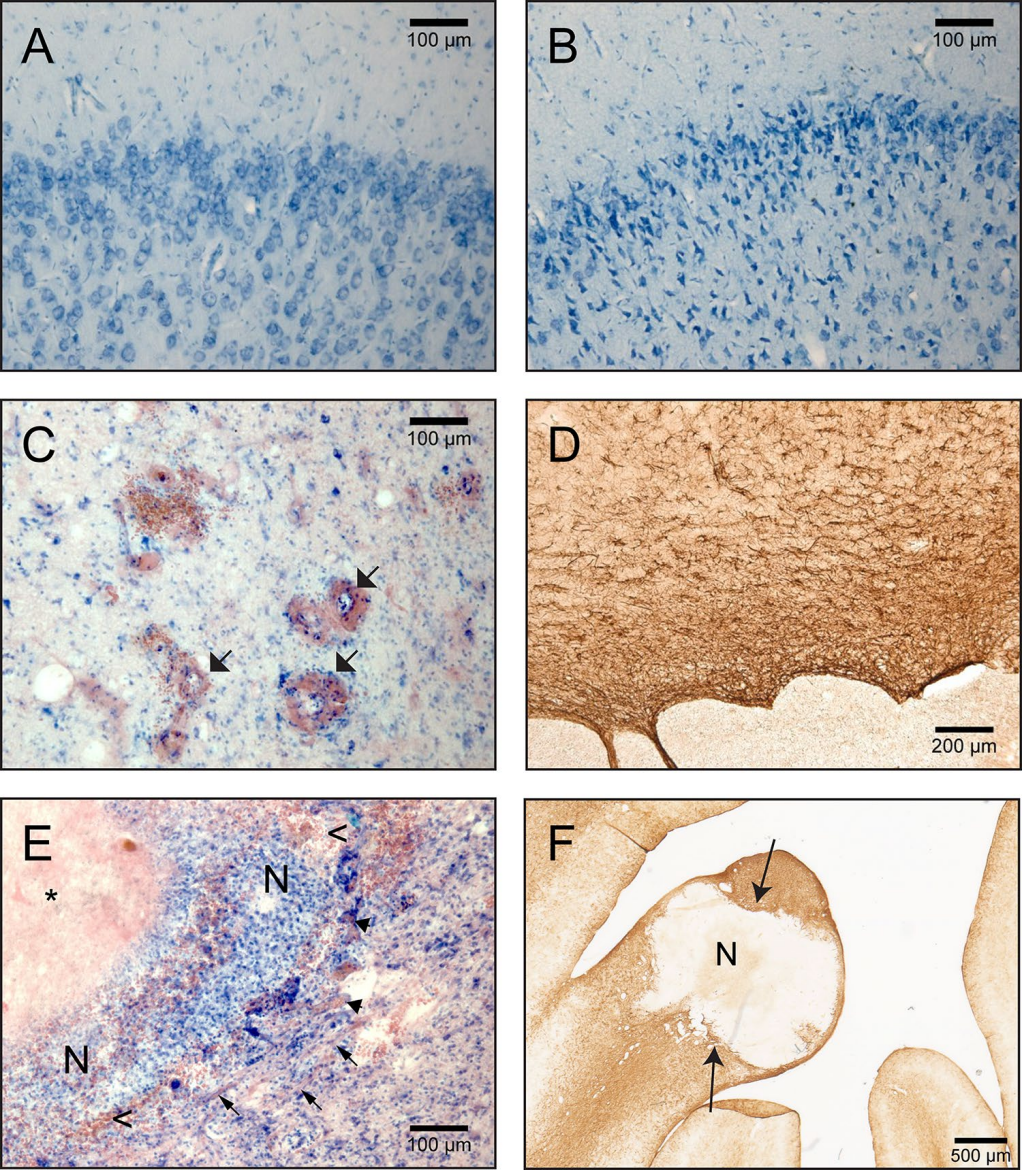
(**A**,**B**) Fragments of the brain sections containing the motor cortex (Animal 02/100 Gy, pictures are taken from the same brain section). (**A**) non-irradiated hemisphere, normal tissue appearance; (**B**) irradiated hemisphere, visible numerous cells with condensed chromatin indicating possible cell physiology changes caused by radiation; (**C**) blood vessels dilatation and thickening (arrows) near the irradiated areas. Section from the minipig A04/80 Gy, Nissl&Eosin; (**D**) glial scar surrounding necrosis (GFAP positive astroglia); (**E**) A04/80 Gy brain sections stained N&E; arrows point to areas with fibrous tissue, arrowheads points to blood vessels. *amorphic, eosinophilic substance (caseous necrosis); n—necrosis (liquefactive necrosis), with numerous white blood cells visible; < —red blood cells in the tissue; (**F**) glial scar (arrows) surrounding the necrosis (N).

Motor cortex radiation-induced histological changes were observed only in the brain A03/100 Gy, which had received 100 Gy via a 5 mm aperture. In this area, tissue necrosis was present, surrounded by reactive astroglia and microglia cells (which formed “dispersed” glial scar). Some cells (single cells within the necrotic area) were positive for Caspase-3 as an indication of apoptosis (Fig. 3C). Some astroglia cells positively stained with Caspase-3 were also observed, which surrounded the area affected by radiation. Around the cortical target, we observed a weak Luxol fast blue (LFB) staining indicating some degree of demyelination. In the remaining animals, no certain radiation-induced histological changes were found. A dispersed glial scar in the motor cortex of animal 03/100 Gy with a 5 mm aperture was observed as a sign of necrotic changes (Fig. 3D). In some of the animals (A02/100 Gy and A04/80 Gy) subtle alterations indicating possible radiation-induced brain change were seen; e.g. “dark neurons” (basophilic) in A02/100 Gy (Fig. 4A,B). Sites with dying cells present (“ghost neurons” in A04/80 Gy) were also observed, but similar changes in neurons were also present in the contralateral hemisphere. Subtle changes in the blood vessel appearance were also noted collaterally. Overall, besides the brain A03/100 Gy/5 mm, there were no obvious light microscopic necrotic changes (Fig. 3A,B,D). The tissue in the control brain (0 Gy) was normal. There were no alterations in the brain tissue in both Nissl&Eosin (N&E) and LFB stainings nor on the brain sections stained with immunohistochemical methods (Cas‐3, GFAP, and Isolectin).

### Histology of white matter

**Figure 5:**
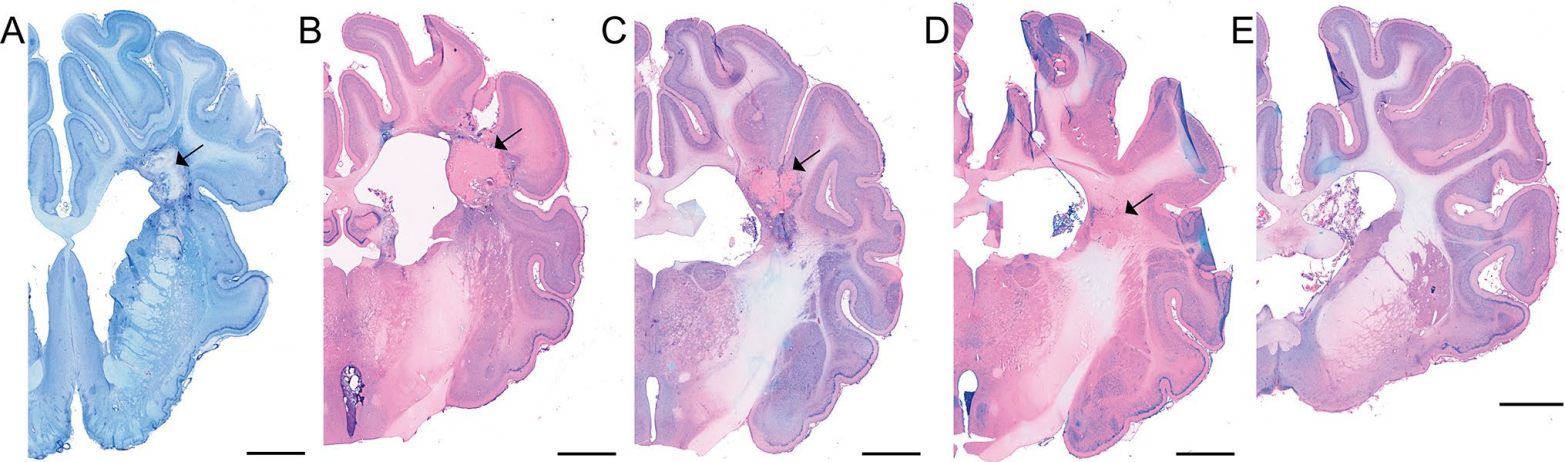
Coronal sections of the brains containing radiation injury in the capsula interna. Visible tissue necrosis and various degrees of inflammation and lesion size. Nissl&Eosin; arrows—necrotic areas (**A** 100 Gy, **B** 100 Gy, **C** 80 Gy, **D** 60 Gy, **E** 40 Gy) scale bar = 5 mm.

Dose–effect relationship in white matter was shown in the histology (Animal 02–Animal 05, Fig. 5A–D). In the brain of the animal that received the lowest dose of radiation (40 Gy), we observed no certain changes (Fig. 5E). The general appearances of the brain tissue of the remaining animals (A02/100 Gy–A05/60 Gy) were as follows: In the center of the target the amorphic, eosinophilic mass was formed (caseous necrosis; in A02/100 Gy almost not visible, Fig. 4E). The amorphic substance was associated or surrounded by tissue necrosis (liquefactive necrosis, largest extent in the A02/100 Gy, and an only small area in A05/60 Gy; Fig. 4E). Necrosis was associated with vascular injury (indicated by the presence of the malformed blood vessels, vascular dilatation and thickening of the vessels walls, hemorrhaging, thrombosis, and frequent red blood cells present in the tissue outside the blood vessels; Fig. 4C). Tissue necrosis following irradiation was associated with varying degrees of inflammation, indicated by the presence of white blood cells including macrophages, activated microglia cells, and formation of the dispersed glial scar around necrotic areas (Fig. 4D,F). A “gradient” of microglia and astroglia reaction was present (amoeboid microglia/macrophages within the necrosis, activated microglia around the necrosis, normal microglia cells in some distance from the necrosis). GFAP (astroglia cells marker) positive cells were visible and decreasing in intensity with distance from the necrosis (Fig. 4F). The thickness/density of the glial scar also decreased with the distance from the radiation injury (Figs. 4F, 5A–D). The appearance of the microglia and astroglia more distant from the radiation target (e.g. in the left hemisphere) was normal. In the brain of A03/100 Gy, some of the macrophages contained a brown deposit (hemosiderin) indicating bleeding in the brain parenchyma and possible disruption of the blood–brain-barrier (BBB), and erythrocytes present in the brain tissue, Fig. 4C,E). In some cases, e.g. in A02/100 Gy, the inflammatory reaction was spreading along the white matter track, both above and below the necrotic area. To some degree, this effect was visible also in A03/100 Gy (Fig. 5A,B). As visualized with GFAP ab and Isolectin, glial cell response was quite limited in the brains A04/80 Gy and A05/60 Gy. No thick layer of astroglia scar was formed; only enhanced astroglia staining and formation of “dispersed” glial scar around necrotic areas were visible. In A05/60 Gy, macrophages/amoeboid microglial cells were present only in close relation to necrosis and within it. The activation of the microglial cells was visible only at a limited distance from necrotic areas. Features characteristic of radiation tissue changes in this experiment were thickening and dilatation of the blood vessels walls, and malformation of the blood vessels, especially in areas close to necrotic tissue (Fig. 4C). We also observed a thickening of the ventricle wall and ependymal layer disruption/enlargement in the ventricle wall adjacent to radiation necrosis. Tissue swelling was also present within irradiated areas. In some cases, (A02/100 Gy and A03/100 Gy, to some degree also A04/80 Gy), the swelling of the brain tissue in the WM was quite large and pressed on the lateral ventricle wall, forming a bulge visible on histological sections (Fig. 5A–C). White matter, visualized by LFB staining, was disrupted in the irradiated areas. Some alterations in the white matter were visible also in areas adjacent to the necrotic zones (Fig. 4F).

## Discussion

Three types of radiation-related side-effects in the brain are identified based on the time of presentation: acute (during or shortly after radiation), subacute or early-delayed (typically up to 12 weeks after radiation), and late (months to years after completion of radiation)^24^. The late effects are characterized by leukoencephalopathy, radionecrosis, and other vascular lesions, such as lacunar infarcts and parenchymal calcifications^25–27^. These delayed radiation-induced neurotoxicities develop and evolve from 3 months to years following treatment and are considered irreversible and continuously progressive^28^. The incidence of radionecrosis depends on the fractionation, the delivered doses, the duration of the follow-up period, the location and volume, and administration of chemotherapy^27^. Two models have been proposed to explain cerebral radionecrosis: the vascular injury theory and the glial injury theory^29^. However, the basic cellular mechanisms of functional radiosurgery are not fully understood despite advances in the usage of focal cerebral irradiation in the treatment of functional brain disorders^15,17^. The latter calls for further investigation of the radio-neurotoxicity in small volumes to advance the functional indications of brain radiosurgery.

In our study, we found irradiation-evoked vascular changes on the MRI and histological necrotic changes in the grey matter (motor cortex) of the animal irradiated with 100 Gy with an aperture size of 5 mm. The necrotic tissue was surrounded by activated microglia and astroglia, which indicates an inflammatory process. MRI changes were most pronounced in the white matter compared to the motor cortex. Lack of systematic changes on PET could be explained by the low spatial resolution of PET compared to the irradiation target size, and/or differences in anesthesia level or duration. However, in line with histology and MRI results, it was possible to detect asymmetry in the PET scans of both animals irradiated with 100 Gy in the white matter and just in the cortex of A03/100 Gy/5 mm aperture. This result supports the finding of other studies, which demonstrated that the FDG-PET standardized uptake values (SUV) ratio at the targeted M1 was decreased compared to the pre-radiation baseline measurements for animals receiving 60 Gy or higher, whilst the lesional effects were only seen after 120 Gy^30^. According to the volume effect of radiation, increasing the volume of the aimed target is supposed to lead to more pronounced tissue damage^31^. In contrast, the same radiation doses with a wider aperture (7.5 mm) in A02/100 Gy resulted in visible changes (condensed chromatin in the cell body of the neurons stained with N&E), without any changes in PET or MRI scans. The dark neurons occur in the early stages of neural injury due to ischemia, hypoglycemia or epilepsy, but they also reflect altered intracellular physiological conditions such as mild metabolic perturbation of the neurons, trans-synaptic stimulation, and release of glutamate, or inhibition of the Na^+^/K^+^ ATPase pump, and not necessarily apoptotic changes^32^. This observation may suggest some ongoing changes in the irradiated area. However, dark neurons can also be considered as artifacts especially in this study since they were observed in both irradiated and unirradiated sides of the brain^33^. To understand how the changes develop, a longer monitoring period with more subjects is needed. The different responses in the two animals to the same radio-dose could be due to the variations in the timing of the radiation damage manifestation development or small geometrical uncertainties stemming from image registration between CT and MR for target definition and accuracy of the image-guided treatment delivery. However, the stochastic effect of the radiation could be mentioned as another interfering factor. Further investigation in the same condition is needed to compare the results.

In this study, we found reactive astrocytes positively stained with the Caspase 3 antibody. Some studies have shown that Caspase 3 beyond its classical role as the main executioner in apoptosis, participates also in cell proliferation, differentiation, and cell cycle regulation^34^. Accordingly, apoptosis happens only in few astrocytes after traumatic, ischemic, and excitotoxic brain injuries^35,36^. These findings allow us to hypothesize that the Caspase 3 positive astrocytes observed in our study do not undergo apoptosis but may play other above-mentioned roles in the brain.

No clear necrotic alteration in the grey matter was seen in the animals, which received lower doses between 80 and 40 Gy, indicating the cortical necrotic effect of radiation in this porcine model.

Radiation evoked necrotic changes in the subcortical white matter in a wider range, from 100 to 60 Gy, but not with 40 Gy, demonstrating lower verge for necrotic effect in white matter. This finding alike other studies confirms that white matter is more vulnerable to radiation-induced changes than grey matter^37–39^. The biggest change was seen in the A03/100G/7.5 mm aperture, which is in line with the volume effect theory of irradiation. The low number of subjects in the current study prevents us from performing statistical analysis. For fractionated radiotherapy with dose per fraction < 2.5 Gy to clinical targets, radionecrosis occurs with an incidence of 5% and 10% at a BED (calculated on alpha/beta of 3) of 120 Gy (range, 100–140) and 150 Gy (range, 140–170) respectively [corresponding to 72 Gy (range, 60–84) and 90 Gy (range, 84–102) in 2-Gy fractions]^40^. For daily fraction sizes > 2.5 Gy, there is less evidence of the incidence and severity of radionecrosis. For single fraction radiosurgery, there is a clear correlation between the target volume and the risk of radionecrosis. The latter increases rapidly once the volume of the brain exposed to > 12 Gy is > 5–10 cm^40^. In the present study, the radiosurgery was given with ultra-high single doses to pig brain with a BED of 570 Gy (BED of 40 Gy × 1) to more than 3000 Gy (BED of 100 Gy × 1) assuming an α/β of 3 Gy. This ultra-high radiation doses resulted in relatively moderate tissue reactions in the brain because of the small irradiated volumes and demonstrates the pronounced volume effects of brain tissue^41^. For this study, sub-lethal repair effects were not considered in the BED calculations. Further studies are warranted to investigate if a relationship between factors such as total dose, treatment time, instantaneous dose rate, and biological repair effects could affect the BED^42^.

The reactions in the white matter were more prominent than the cortex, which was also demonstrated by Calvo et al.^43^. The reason for the higher radio-sensitivity of the white matter is still unknown. Considering the vascular endothelial injury as the primary mechanism of injury, it has been hypothesized that differences in vascular supply and density could be a possible cause of dissimilarities in susceptibility of the white and grey matter to radiation^39,44,45^. In theory, the higher regional blood flow and metabolic rate in the grey matter could potentially make it more sensitive to irradiation. The dividing cells in the brain (e.g. glial cells) have traditionally been considered more vulnerable to radiation than the non-dividing cells (neurons)^19,46^, although this theory has been questioned in recent studies^47^. Likewise, the density of glial cells and oligodendroglial progenitor cells is higher in white matter^48^. Concerning the two mentioned factors, the other suggested pathway of the lesional effect of radiation, namely, the progressive loss of glial cells seems to be more relevant to explain the higher radio-sensitivity of white matter. Accordingly, radiation-induced DNA damage might be expected to have a greater effect upon the axonal side rather than perikaryon because of disruption in the division process of surrounding oligodendrocytes producing myelin sheaths of the white matter tracts.

Another observation regarding the white matter was the gradient of cellular reaction to the radiation with the distance from the irradiation center, changing from complete liquefactive necrosis in the core to activated microglial cells around it (sub-necrotic area) and finally the normal tissue in distance; as suggested by Regis as the Cockade dose-dependent effect to radiation^49^. Accordingly, the adjacent white matter (the sub-necrotic area close to irradiated cortex) in A03/100 Gy, had also shown fiber changes/disappearance visualized by LFB as probable evidence of dose–response by itself^19,50^. On the other hand, it is not possible to rule out the effect of intercellular messengers, since we have also observed characteristic radiation-induced changes such as thickening, dilatation, and malformation of the blood vessels walls, as well as ependymal layer disruption/enlargement in ventricles. These changes were seen especially close to the necrotic tissue in the white matter, which is in-line with the bystander effect of the irradiation^51^. Furthermore, the spreading inflammatory changes along the neural tracts in the white matter of A02/100 Gy–A04/80 Gy suggest migration of inflammatory messengers, which may play a role in the distant post-irradiation changes. This idea is, however, based on some other studies showing over-expression of alpha tumor necrosis factor (TNF-α) and transforming growth factor—Beta 1 (TGF-β1) in both irradiated and un-irradiated hemispheres of rat brains after radiation^52^. Due to the small size of the rat brain, the contralateral side considerably receives a significantly high amount of radiodoses, which may affect the results, and mandates further investigation in the large animal. However, the same observation was also noted in cultured monocytes taken from patients who have undergone radiosurgery^53^. Otherwise, the disintegration of the entire neuron with its axonal branches inevitably follows Wallerian degeneration towards the tip of the axon and retrograde towards the cell body, which can also be mediated by inflammatory messengers^54^. Taken together, these findings theoretically might point at the post-irradiation roles of cytokines in the brain tissue.

With the use of 5–7.5 mm aperture and a follow-up of 7 months, we found the radiation doses causing necrotic changes in both grey and white matter in a minipig model of stereotaxic brain radiosurgery. Moreover, gradients of changes were observed indicating a dose-dependent reaction to the radiation. Alteration in cellular features in adjacent tissue and further from the irradiated area without necrotic changes, indicating biological alterations, may be related to the neuroinflammatory messengers according to the other studies^14,16,18–20^; however, they could simply be a reaction to the neighboring necrosis.

The presented large animal model may facilitate further investigations on the effect of radiosurgery by considering the radiation doses, the volume, and type of the brain tissue, whether the effect may be based on ablation or biological alterations in the brain tissue without causing necrotic injury. To clarify the biological responses to the radiation in acute, subacute and chronic phases, further electrophysiological studies, advanced MRI technics like perfusion/Diffusion or FLAIR sequences, and histological studies at different time points with more animals in a longer period are still needed as a necessary step before any clinical usage of this method.

## Methods and materials

Six female Göttingen minipigs, weighing averagely 15 kg, aged four to six months were used in this study. The Danish Animal Experiments Inspectorate (2016-15-0201-01103) approved this study in compliance with the ARRIVE guidelines and the 2010/63/EU directive for animal experiments. One animal (A01) was used as the control, and five animals (A02–A06) received various doses of photon radiation on two cerebral sites: (1) Left primary motor cortex (M1-grey matter), and (2) Right subcortical white matter (WM) (Table 2: Animal/Treatment Allocation Table).

**Table 2:** Animal/treatment allocation table.

Animal number	Left: dose/aperture/target	Dose to 0.034 cc^b^ (Gy)	Treatment time/min	Maximum point dose (Gy)	Right: Dose/aperture/target	Dose to 0.034 cc (Gy)	Maximum point dose (Gy)	Treatment time/min
Control animal	0 Gy/NA^a^/NA				0 Gy/NA./NA			
Animal 02	100 Gy to 0.111 cc/7.5 mm/M1	109.4	41	114.0	100 Gy to 0.040 cc/5 mm/IC	102.8	120.3	47
Animal 03	100 Gy to 0.042 cc/5 mm/M1	103.4	47	120.6	100 Gy to 0.108 cc/7.5 mm/IC	109.1	113.9	38
Animal 04	80 Gy to 0.041 cc/5 mm/M1	82.5	36	96.8	80 Gy to 0.041 cc/5 mm/IC	82.3	96.6	38
Animal 05	60 Gy to 0.040 cc/5 mm/M1	61.6	29	72.2	60 Gy to 0.040 cc/5 mm/IC	61.6	72.2	32
Animal 06	40 Gy to 0.042 cc/5 mm/M1	41.3	22	48.3	40 Gy to 0.041 cc/5 mm/IC	41.2	48.3	23

^a^Not applicable.

^b^0.034 cc corresponds to a sphere with a diameter of 4.0 mm.

Two weeks prior to radiation, all animals underwent a baseline stereotactic structural MRI (T1 and T2-weighted image series) and FDG-PET/CT scan. The post-irradiation follow-up imaging procedures are shown in Table 3.

**Table 3:** Schedule of procedures.

Schedule of procedures	
1 week prior to radiosurgery	Fiducial screw placement
Day of radiosurgery	Planning CT, planning MRI, baseline PET
1 and 3 weeks post-rad	MRI with Gad, PET
3 Months post-rad	MRI with Gad, PET
5, 6 and 7 months post-rad	MRI with Gad, PET. Killing, Histology

All the imaging and radiation procedures were conducted under general anesthesia. Animals were pre-medicated with 0.8 mg/kg midazolam and S-ketamine (20 mg/kg) IM. Ear veins were catheterized (21G venflon) and anesthesia was induced with a second IV injection of Midazolam/S-ketamine. Shortly afterward, the minipigs were intubated (6.0 mm-tube) and anesthesia was maintained using approximately 2.1% isoflurane through mechanical ventilation. A mobile pulse-oximeter device was used to constantly monitor the oxygen saturation and heart rate during all the procedures except the MRI, during which the expiratory carbon dioxide was controlled by the anesthesia machine and the pulse by MRI-compatible pulse monitoring device. Prior to fixation in an MRI-compatible stereotaxic localizer box, 1.25 mg/kg of 0.5% bupivacaine hydrochloride (Marcain, AstraZeneca, DK) was injected subcutaneously over the frontal and zygoma bones at the sites of the surgical incision and localizer box fixation pinholes^55^. As a stereotaxic reference point, a copper-sulfate filled fiducial marker was placed in a drill hole in the skull close to bregma^56^.

Animals underwent a 3D T1-weighted MRI brain scan (3.0 T Siemens Skyra) with and without contrast (Gadoteric acid 62 µg/kg (Dotarem)) with 1 × 1 × 1 mm voxels, Time to Echo 3.7 ms, Repetition Time 2200 ms, inversion time 960 ms, Fractional anisotropy 9 deg., 256 × 256 mm image size. During the 10 min MRI acquisition, animals were kept anesthetized with Propofol (2–5 mg/kg/min), and hand-ventilated.

### Radiosurgery treatment planning and treatment delivery

**Figure 6:**
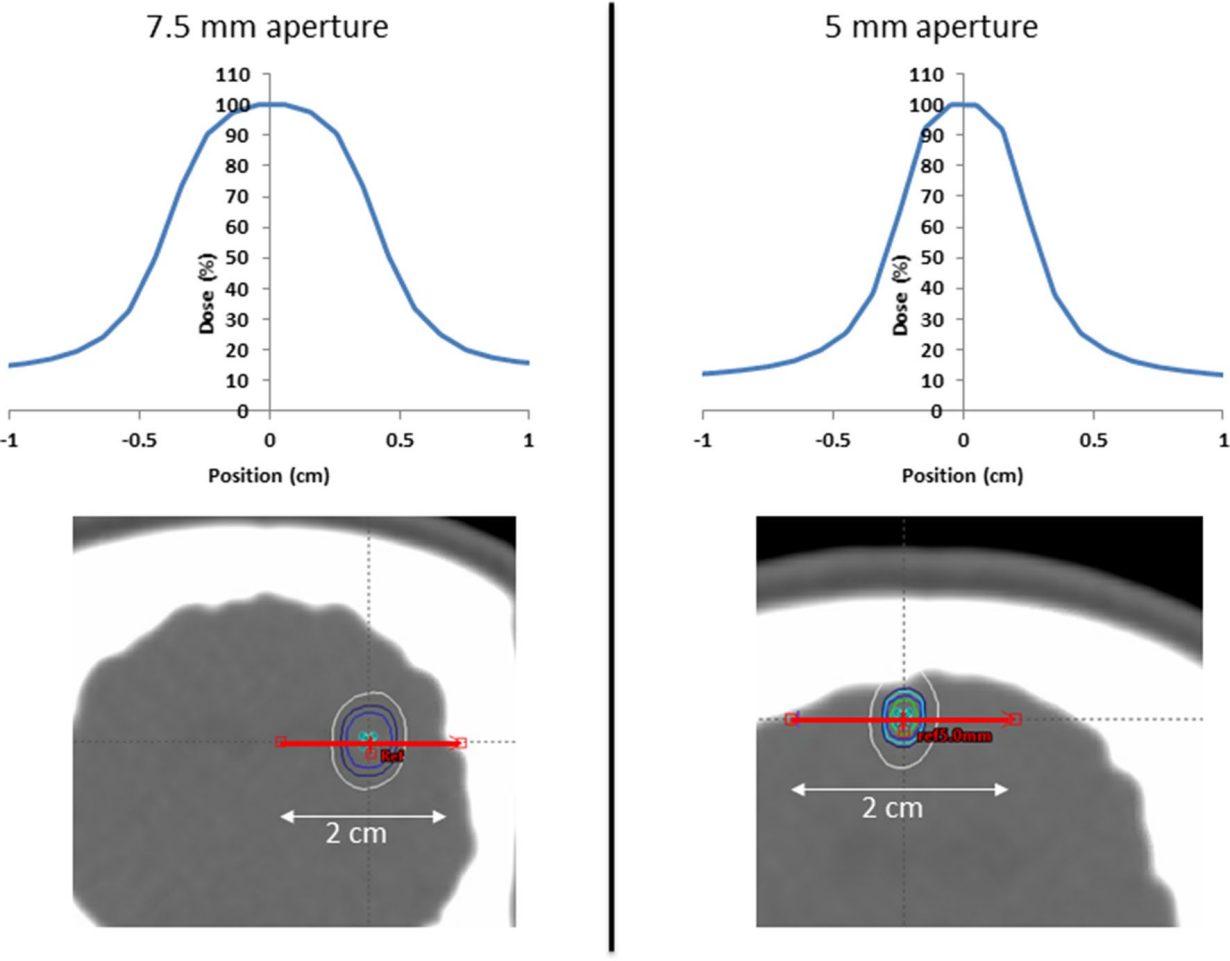
Dose profiles for Animal 03/100 Gy centrally through the targets along the red line. 100% dose corresponds to the maximum point doses of 120.6 Gy (5 mm aperture, Motor cortex) and 113.9 Gy (7.5 mm aperture, White matter). Isodose lines of 60%, 80%, 90%, and 100% are shown around the targets.

SRS planning was based on fused CT and MR image data sets. The planning CTs were acquired with 1.5 mm slice thickness (Brilliance Big Bore CT, Phillips, Amsterdam, The Netherlands). The T1 MR scans were acquired with a 1.0 mm slice thickness. Two-point targets were defined in: (1) the primary motor cortex (M1-grey matter), and (2) the subcortical white matter, in the centrum semiovale and upper part of capsula interna. Treatment was planned using the Eclipse treatment planning system (Eclipse 13.7, Varian Medical Systems, Palo Alto, CA, USA) and based on a Truebeam linear accelerator equipped with a High-Definition 120 multi-leaf Collimator (MLC) of 2.5 mm leaf widths. Each treatment plan was based on 15 conventional MLC defined non-coplanar fields with quadratic aperture sizes of 5 × 5 mm or 7.5 × 7.5 mm (in the isocenter plane), centered around the target point. The target-point normalized doses calculated by the Eclipse treatment planning system (AAA v. 13.7.14 dose calculation algorithm with calculation grid size 0.1 cm) were corrected with small-field dosimetric factors obtained from measurements with a diamond detector (Natural diamond detector, type 600003 (PTW Freiburg GmBH)) in a water phantom. Target max doses ranged from 48.3 to 120.6 Gy (Table 2). The beam energy was 6 MV and each beam was delivered with a dose rate of 600 MU/min. This corresponded to a dose rate during field delivery of 3.9–4.2 Gy/min (7.5 mm fields) and 3.4–3.8 Gy/min (5 mm fields) at dose maximum centrally in the target. The total treatment times per lesion including breaks for gantry rotations and couch rotations between treatment fields varied between 22 and 47 min. Localization of each target for radiation was obtained by fusing a pre-treatment 1.5 mm slice thickness kV cone-beam CT (CBCT) scan with the planning CT, using the onboard imaging system of the Truebeam accelerator. Couch corrections were performed according to the CBCTs with 4 degrees of freedom (translational and rotation around the vertical axis). This provided sub-mm setup accuracy of each target relative to the radiation beams (based on internal QA measurements). Dose profiles for animal A03/100 Gy/5 mm aperture are shown as an instance in Fig. 6.

### FDG-PET/CT and MRI analysis

FDG-PET/CT scans were aligned to the pre-irradiation CT scans using PMOD Imaging Software v3.7 (PMOD Technologies). Co-registration of CT (Fig. 2B) and PET images was performed. Similarly, the fusion of MRI and PET images were done (Fig. 2C,D). Following 3-D reconstruction and normalization, voxel intensity change from baseline was determined by digitally subtracting of the two data sets. Volumes of interest were included in each irradiated target volume. PET images were analyzed afterward to find metabolic alterations in the targeted areas. MRI images were visually inspected for evidence of lesions associated with the target zones to determine whether evidence of tissue changes was seen. The volumes of the visible brain changes were calculated by the use of Cavalieri volume estimation and ImageJ.

PET data were analyzed by two different research centers to avoid bias.

### Histology procedures

**Figure 7:**
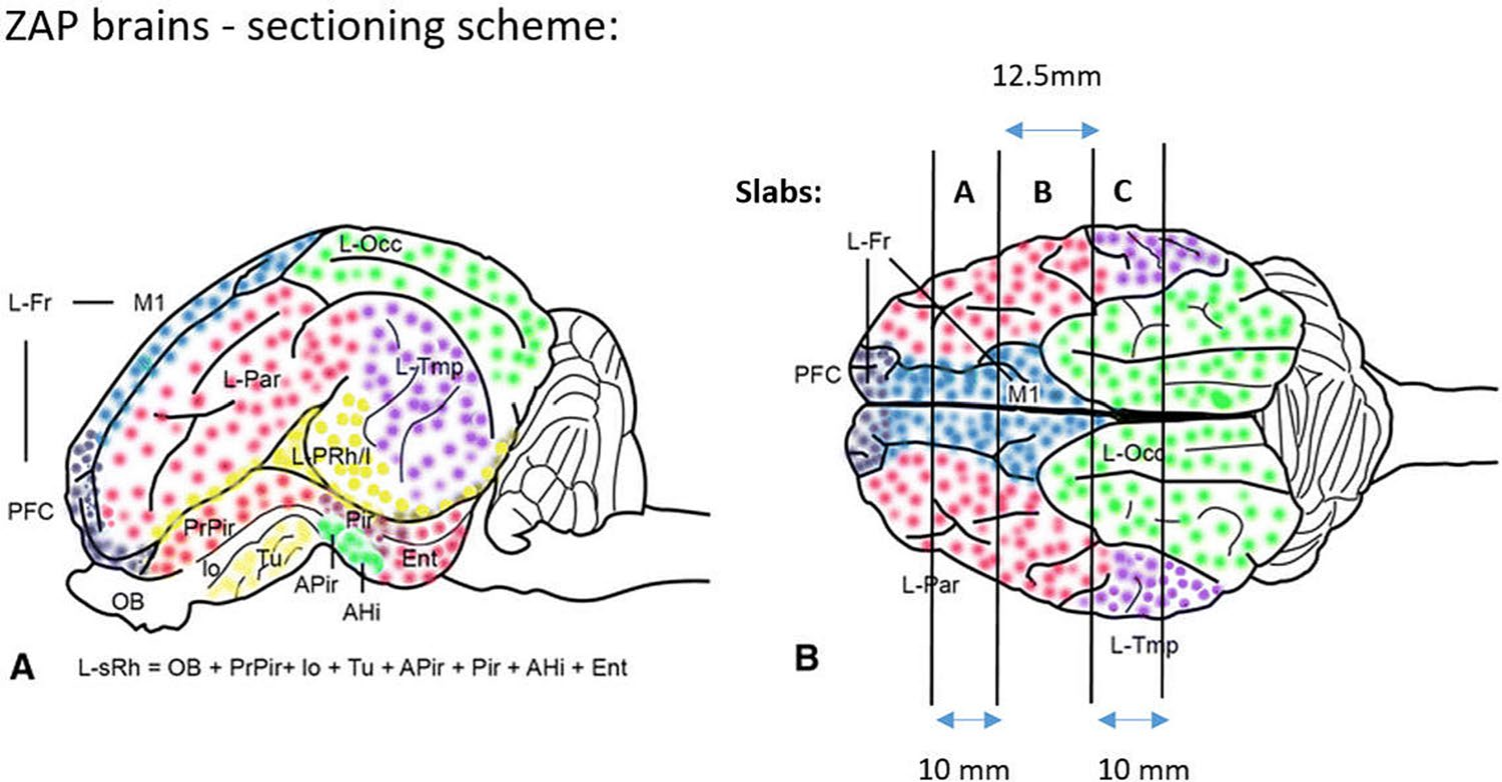
Brain sectioning (modified) Reprinted by permission from Springer, Brain Struct Funct Bjarkam C. et al. July 2017, Volume 222, Issue 5, pp 2093–2114.

Approximately six months after irradiation the animals were killed by an overdose of Pentobarbital (400 mg/mL) and transcardially perfused with 5 L of 10% buffered formalin^57^. The brains were then removed and post‐fixed for five days in the same fixative and then sliced into 1.25 cm thick coronal tissue slabs. The tissue slabs containing areas of interest (Fig. 7, slabs A, B, and C) were cryoprotected in a 30% sucrose solution in buffered saline (PBS) for 7–10 days, followed by freezing in isopentane cooled by dry ice (Fig. 7). The frozen brain slabs were then cryostat sectioned into 40 µm thick sections, which were either directly mounted on the microscopic slides or preserved free-floating in DeOlmos cryoprotecting solution. To assess the tissue reaction, the following staining methods were used: N&E to show general changes in the tissue; LFB for visualization of the white matter; Caspase-3 (Cas-3) antibody to visualize apoptotic cells; GFAP antibody to visualize astroglial reaction and Isolectin (BSI‐B4) to visualize microglia and macrophages. The immunohistochemical stainings were performed as followed: Astroglial cells were visualized according to the Avidin–Biotin Complex protocol; free-floating sections were incubated in 48C overnight with mouse monoclonal anti-GFAP antibody (Abcam [Cambridge, UK], #ab4648) diluted 1:2000 in 0.05 M TBS with 1% Triton X-100 and 0.2% milk followed by incubation with secondary antibody (diluted 1:200 goat anti-mouse, Abcam #ab6788). The reaction was visualized using 0.1% DAB with 0.3% H2O2. Caspase-3 was visualized according to the above protocol with primary antibody diluted to concentration 1:600 (rabbit polyclonal Ab, Abcam, #ab13847) and secondary ab diluted to concentration 1:200 (biotinylated goat anti-rabbit ab., Abcam, #ab6720). To visualize macrophages and microglia cells the sections were incubated for 5 h with Isolectin B4 peroxidase conjugate (Sigma-Aldrich L5391) diluted to the concentration 5 mg/mL and developed using DAB reaction. The sections were dehydrated with 99% ethanol followed by xylene and coverslipped using Pertex. The qualitative description was based on approximately 800 brain sections in total.
